# Psychoanalytic contributions in distinguishing willful ignorance and rational knowledge avoidance

**DOI:** 10.3389/fpsyg.2023.1025507

**Published:** 2023-02-14

**Authors:** Christopher Kam

**Affiliations:** School of Counselling, Psychotherapy and Spirituality, Faculty of Human Sciences, Saint Paul University, Ottawa, ON, Canada

**Keywords:** self-deception, willful ignorance, knowledge avoidance, psychoanalysis, ego inflation

## Introduction

Discussions on self-deception have occurred in academia for a long time (Deweese-Boyd, 2021). Part of the difficulty in defining self-deception is the puzzling nature of how an individual can seem to be both aware and unaware of tricking oneself at the same time (Lewis, 1996). The Stanford Encyclopedia of Philosophy notes that “self-deception involves a person who seems to acquire and maintain some false belief in the teeth of evidence to the contrary as a consequence of some motivation, and who may display behavior suggesting some awareness of the truth” (Deweese-Boyd, 2021). In the midst of exploring the nature of self-deception, there is an attempt to distinguish between willful ignorance and rational knowledge avoidance since the latter can be useful, arguably beneficial in some cases (Arfini and Magnani, 2021) and can assist in overcoming self-deception. Although the nuanced distinction between willful ignorance and rational knowledge avoidance is interesting and helpful, there are gaps explaining the different psychological processes occurring in one compared to the other. Some of these explanatory gaps are in the unconscious dimensions of the mind. Here, psychoanalytic contributions that outline unconscious processing can offer additional explanatory power in making sense of how rational knowledge avoidance can sometimes be prudent while conceptually contrasting it with the imprudence of willful ignorance.

## Willful ignorance vs. rational knowledge avoidance

In the research literature, willful ignorance has an all-encompassing quality of “the general avoidance of situations that let someone aware of certain information, evidence, or knowledge” (Arfini and Magnani, 2021, p. 4). In contrast, knowledge avoidance can be rational and relatively specific in avoiding certain information for particular reasons. Arfini and Magnani (2021) write “we can say that people are willfully ignorant of something when they avoid all circumstances that would allow them to acquire that knowledge, even by accident. Instead, people in a condition of knowledge avoidance do not perform the necessary steps to get a specific piece of information” (p. 3). The latter involves avoiding the knowledge of certain information that may emotionally affect one's judgment or reasoning. People who are engaging in knowledge avoidance are “well aware of which information they are avoiding and why” (p. 4–5). It is similar to the notion of rational ignorance, which happens “When the costs of acquiring knowledge outweigh the benefits of possessing it” (Williams, 2021, p. 7,807).

## Psychoanalytic contributions to the distinction

This distinction between willful ignorance and rational knowledge avoidance can benefit from insights from the psychoanalytic tradition of psychology that focuses on unconscious processes. Boag (2017) notes that “a mental process is descriptively unconscious if we are presently unaware of it. For example, a belief would be described as descriptively unconscious if it was believed, without the person currently being aware of having the belief” (p. 2). He writes elsewhere that there is a difference between cognitive science's “cognitive unconscious” and the psychoanalytic “dynamic unconscious” (Boag, 2020). The former has non-motivated obstacles to becoming aware of something (e.g., automated processing that is implicit but not presently available to the mind while no motivated repression is involved). The latter has motivated obstacles preventing awareness (e.g., unconscious repression involving defense mechanisms defending against unprocessed pain) (Solms, 2017, 2018; Boag, 2020).

Empirical research shows significant effects of unconscious influences on conscious processes. For example, emotions can be experienced in brain regions without one's conscious awareness (Brooks et al., 2012), subliminal pictures can prime people to influence their narratives unconsciously (Kawakami and Yoshida, 2014), and unconscious influences can affect behaviors and goals without conscious initiation (Bettiga et al., 2017). In addition, there is support showing the difference between one's explicit self-concept and implicit self-concept and that discrepancies between the two are related to psychological suffering (Bosson et al., 2003; Zeigler-Hill and Terry, 2007; Fabbro et al., 2017). For example, implicit self-concept, measured by reaction times to words or ideas, may or may not relate to a person's more explicitly expressed self-concept (Greenwald et al., 2002). Also, clinical experiences in Short-Term Dynamic Psychotherapy (based on psychodynamic principles), show how the lifting of unconscious defense mechanisms such as repression can result in unconscious content emerging that has been unprocessed (Davanloo, 1987; Abbass et al., 2012; Town et al., 2013; Johansson et al., 2014).

## The relevance of ego inflation

Carl Jung, one of the leading psychoanalysts of the twentieth century, defined ego inflation as an unconscious expansion of one's personality beyond its proper limits. When this happens, a person identifies with a persona or archetype that produces an exaggerated sense of one's self-importance and is usually motivated by feelings of inferiority (Jung, 1934–1939, 1963; Schlamm, 2020). This provides psychoanalytic commentary on one version of self-deception where a person has a dispositional tendency to have a positive self-image that is unrealistic (Sackeim, 1983). When an ego is inflated, the person views themself as better than everyone else (Helander and Andersson, 2014). This can lead to the person's ego feeling easily pricked, resulting in retaliation against perceived offenders (Bushman and Baumeister, 1998; Neff, 2011). Ego inflation can be motivated by an inferiority complex resulting from psychic fragments of painful inferiority in the mind that have been split-off from conscious awareness due to previous traumatic influences (Jung, 1970). When this happens, a person can feel inferior with low self-worth in the unconscious parts of the mind, namely the “shadow.” Here, inferior traits of character that the individual refuses to acknowledge never fully go away; on the contrary, they are continually trying to thrust themselves onto the person's conscious mind (Jung, 1969). Due to the repression, the person has an indirect but even stronger desire for affirmation that is overcompensating and exaggerated in its hunger. This will lead to a pursuit of ego-inflation, where one may seek flattery from others to feel energized by a socially reinforced shiny persona or a thickly bright self-archetype. This self-archetype contains an image of oneself that seems, in everyday language, perfect in symmetry, flow, crispness, and thickness of being. Here, the ego inflation temporarily drowns out the pain from one's unconscious inferiority complex. Such a process, like an addiction that can increase one's propensity toward episodes of willful ignorance, may lead one to feel ecstatic for a moment but dark, heavy and empty after. This is consistent with research that shows that the inflated ego is unstable (Kernis et al., 1989) and constantly threatened by doubts (Jordan et al., 2005).

## An illustration of the integration

All this conceptualization can be tied together in an illustration consistent with research showing that the rise of social media inadvertently encourages ego inflation (Jordan et al., 2014). Pretend there is a young adult named Suzy. Suzy, through an extended journey of self-discovery and awareness of her past patterns, is aware of her propensity to fall victim to a certain type of ego inflation, namely flattery from social media feedback for her online posts. She realizes that this tendency is due to an inferiority complex formed from her past, where she felt low self-worth and tried to compensate for it by manipulating situations to elicit flattering compliments. She realized that she was never satisfied with flattery for long and always needed more. She is aware that in the recent past, she used to be preoccupied with and addicted to her social media account, specifically by checking how many likes, comments, and flattering pieces of feedback she received from her posts. This would lead to a roller coaster of first identifying herself with a shiny, thickly bright persona or self-archetype that others would reinforce through flattery. This led to an exaggerated sense of self-importance, which eventually led to a need for more flattery, eventual jealousy, and then emptiness from comparing herself with others who ended up seeming more “shiny” in “thick brightness” in social media attention. Depressive and empty feelings would follow the dopamine high. Recognizing this propensity, Suzy now tries to be prudent in her use of social media and has decided for the next few months to try an experiment by not checking any notifications of feedback from any of her social media posts. This is an example of rational knowledge avoidance, since it describes “a condition in which agents avoid some knowledge to refrain from anticipated costs (in terms of pain, anxiety, or regret) of possessing it…In these situations, people avoid acquiring those pieces of information that would impact their emotional state, reasoning abilities, and decisions” (Arfini and Magnani, 2021, p. 6). In this case, Suzy is cognizant of her propensity to relapse into a social media addiction that seeks flattery that is fueled by an inner sense of lack or inner deficiency (Naranjo, 1994). Similar illustrations could be made in this framework of using rational knowledge avoidance to prevent ego inflation with other examples of addictions such as substance abuse, gambling, or alcoholism.

To complete the illustration of this article's integrated concepts, it can be noted that if Suzy decided to indulge in flattering social media feedback without any boundaries or self-control, she would engage in ego inflation which would lead down another path, namely willful ignorance. For instance, if this alternative scenario occurred to the extent where she intentionally ignored and disregarded all constructive criticism given in response to her social media posts, then she would be engaging in willful ignorance, specifically in the form of wishful thinking, which is a positive illusion motivated by believing in one's wishes while avoiding content that is inconsistent with it (Sigall et al., 2000; Mayraz, 2011; Jefferson et al., 2017). Here, Suzy would be psychoanalytically motivated to inflate her ego and identify herself as a shiny and thickly bright persona or self-archetype and be motivated to disregard and become ignorant of any disconfirming information that would challenge her ego-inflated view of herself. The motivation of this type of information avoidance would come from an unconscious desire to compensate for one's feelings of inferiority from one's inferiority complex, which would be the opposite of rational knowledge avoidance. This would last as long as the duration of the spell of ego inflation.

From this integrative illustration, we can see that, from a psychoanalytic perspective, rational knowledge avoidance can occur when a person intends to prevent ego inflation while willful ignorance can occur when a person wants to indulge in it.

## Conclusion

This article explored the distinction between rational knowledge avoidance and willful ignorance through the lens of psychoanalytic concepts, particularly ego inflation. Relevant conceptual and empirical work were outlined in an integrative way that climaxed with an illustration. Future work can be done to study these integrated concepts from an empirical level and see if they hold water in the laboratory and everyday life. Clinical applications can then be made in terms of when knowledge avoidance can be rational and prudent as well as when it can be imprudent, dysfunctional, and detrimental to wellbeing. As it is harder to regulate ego inflation when individuals are unaware of their own underlying dynamics of themselves, more awareness of these dynamics can help (Yilmaz, 2020). Understanding the difference between willful ignorance and rational knowledge avoidance from integrating philosophical concepts, psychoanalytic commentary, and empirical studies can help individuals understand when it is in their best interest to know something and when it is not.

## Author contributions

The author confirms being the sole contributor of this work and has approved it for publication.
